# Oxygen therapeutic window induced by *myo*-inositol trispyrophosphate (ITPP)–Local pO_2_ study in murine tumors

**DOI:** 10.1371/journal.pone.0285318

**Published:** 2023-05-11

**Authors:** Martyna Krzykawska-Serda, Dariusz Szczygieł, Szymon Gaweł, Agnieszka Drzał, Małgorzata Szczygieł, Maciej M. Kmieć, Andrzej Mackiewicz, Claudine Kieda, Martyna Elas

**Affiliations:** 1 Faculty of Biochemistry, Biophysics and Biotechnology, Department of Biophysics and Cancer Biology, Jagiellonian University, Kraków, Poland; 2 Department of Radiology, Geisel School of Medicine, Dartmouth College, Hanover, New Hampshire, United States of America; 3 Department of Cancer Immunology, Greater Poland Cancer Centre, Poznan University of Medical Sciences, Chair of Medical Biotechnology, Poznan, Poland; 4 Laboratory of Molecular Oncology and Innovative Therapies, Military Institute of Medicine -National Research Institute, Warsaw, Poland; 5 Center for Molecular Biophysics UPR 4301 CNRS, 45071, Orleans, France; 6 Department of Oncology, Centre of Postgraduate Medical Education, Warsaw, Poland; East Carolina University Brody School of Medicine, UNITED STATES

## Abstract

Hypoxia, an inevitable feature of locally advanced solid tumors, has been known as an adverse prognostic factor, a driver of an aggressive phenotype, and an unfavorable factor in therapies. Myo-inositol trispyrophosphate (ITPP) is a hemoglobin modifier known to both increase O_2_ release and normalize microvasculature. Our goal was to measure the tumor oxygen partial pressure dynamic changes and timing of the therapeutic window after ITPP systemic administration. Two syngeneic tumor models in mice, B16 melanoma and 4T1 breast carcinoma, were used, with varying ITPP dose schedules. Tissue oxygenation level was measured over several days in situ in live animals by Electron Paramagnetic Resonance oximetry with implanted OxyChip used as a constant sensor of the local pO_2_ value. Both B16 and 4T1 tumors became more normoxic after ITPP treatment, with pO_2_ levels elevated by 10–20 mm Hg compared to the control. The increase in pO_2_ was either transient or sustained, and the underlying mechanism relied on shifting hypoxic tumor areas to normoxia. The effect depended on ITPP delivery intervals regarding the tumor type and growth rate. Moreover, hypoxic tumors before treatment responded better than normoxic ones. In conclusion, the ITPP-generated oxygen therapeutic window may be valuable for anti-tumor therapies requiring oxygen, such as radio-, photo- or immunotherapy. Furthermore, such a combinatory treatment can be especially beneficial for hypoxic tumors.

## Introduction

Anticancer therapies are hampered by hypoxia. This phenomenon is not only associated with the local and momentary low oxygen level in the tissue but also with previous development of a hypoxic, aggressive cancer phenotype encompassing higher invasive and metastatic potential, immunosuppression, and drug resistance [1–3]. The dynamic nature of the tumor microenvironment, with its high pressure, poor perfusion, and quick remodeling of the vascular network, makes hypoxia impossible to defeat. The classical approaches include oxygen breathing [4–6], blood substitutes [7,8], and antiangiogenic treatments [9,10]. Extensive research into tumor hypoxia has resulted in the discovery of temporary tumor vessel normalization [11]. Such normalized, functional vasculature inside the tumor leads to improved oxygenation for a short period of time, creating a “therapeutic window” of improved oxygenation which enhances the efficacy of therapies, such as radio- or phototherapy [12]. The concept of therapeutic oxygen window generation might be helpful for other types of therapies, such as immunotherapy [13] and chemotherapy [14].

*Myo*-Inositol trispyrophosphate (ITPP) has been an effective option. It has been shown to penetrate red blood cells by binding to band 3 and to work as an allosteric effector of hemoglobin, shifting the Hb/O_2_ saturation curve [15]. ITPP has also been shown to stabilize normalized vessels by interacting with and activating endothelial PTEN. This offers an efficient way to stabilize the vessel normalization and increase the length of the therapeutic window [16,17]. Indeed, an approach to lengthen the therapeutic window has been proposed as an alternative to antiangiogenic strategies for their limited normalization effect on the vessels [18]. However, in the recently published report on first-in-patient ITPP treatment, no clear correlation was demonstrated between the dosing or tumor type and the responses [19]. As the authors suggested, this may have been due to the very heterogeneous patient population. This finding lays the ground for our hypothesis about the importance of tumor oxygenation before ITPP treatment.

The process of vessel normalization is thus a critical mechanism that needs to be studied in various settings and throughout the tumor growth and development of its microenvironment. Consequently, one has to find sensors of the evolution of oxygen availability in the tumor site, which are good indicators of the evolution of the treatments. It must report the oxygen partial pressure (pO_2_) value and its evolution along the protocol applications. Such reporters will be necessary in the case of vessel normalization attempts aiming to assess which vessel normalization protocols are valid to design efficient adjuvant therapies. Indeed a punctual but stable sensor of oxygen partial pressure in the tumor site is a tool to follow the efficacy of an antihypoxic treatment along the various protocols of its administration [20,21].

As shown in previous studies, treatment with ITPP in tumor animal models rapidly induces an increase in the pO_2_ inside a hypoxic tumor. While the vessel normalization step could be achieved when injections were repeated, stable normalization remained the main challenge for the success of adjuvant therapies based on the synergistic effect brought to most anticancer treatments by blood flow restoration. The dependence on tumor type was observed among diverse parameters (e.g. a change in the M1 and M2 macrophage-type proportions, increased proportions of NK and CD8+T cells, and a reduction in Tregs and Th2 cells). Thus the follow-up of protocol effects by a long term oxygen sensor is necessary to establish reliable treatment procedures by allowing standardization of the stabilization of the normalized vasculature as a condition prior to chemo-, radio-, and, importantly, immunotherapy [13].

Electron paramagnetic resonance (EPR) oximetry is one of the few oximetric methods applicable *in vivo*. It requires introducing a paramagnetic oxygen-sensitive probe into the tissue and acquiring its EPR signal. One of the strategies involves spatial imaging using soluble probes injected into the animal [22–24]. Another option involves using particulate probes, e.g., LiPC, which enables repetitive pO_2_ readings from the exact location in the tissue [25–27]. This approach was used clinically in pO_2_ measurements in the skin of diabetic patient legs [21,28] and in cancer patients [29]. We have used an oximetric spin probe OxyChip (LiNc-BuO) [30,31]. This is a very sensitive method to provide absolute pO_2_ values in long term experiments. The sensor may be implanted into the tumor tissue or injected together with tumor cells and provides local pO_2_ value from a small area around the probe. Even though the measurement is local, it is representative of tumor oxygenation and responds well to changes such as oxygen breathing [29]. The vital advantage of this approach is high sensitivity at low pO_2_.

Our goal was to estimate the effects of systemic treatment with ITPP by monitoring local pO_2_ in tumors using OxyChip and EPR. To ascertain the timing of the oxygen therapeutic window generated by ITPP, we used various application protocols and two orthotopic immunocompetent murine tumor models, 4T1 and B16F10. We demonstrated that ITPP led to either a transient or sustained increase in tumor pO_2_ and that some hypoxic tumor areas became normoxic, which shows that ITPP systemically mitigates tumor hypoxia. The novelty of our study is not only related to absolute, longitudinal measurements of local oxygen partial pressure but also highlights the relevance of pre-ITPP treatment tumor oxygenation and proper ITPP protocol to reduce tumor hypoxia. The solid-state spin probe OxyChip was previously developed and tested as described by Hou et al. [30]. The application of solid crystal probes is minimally invasive and is well tolerated by the tissues (i.e., no signs of inflammation). The probes also allow the measurement of oxygen partial pressure with excellent resolution from the area around the probe and for a prolonged time. However, several aspects need to be taken into consideration. First of all, it is uncertain how large is the area represented by the measured EPR spectrum, reflecting averaged oxygen partial pressure. The probe position was checked occasionally by ultrasound and always during necropsy, and no changes were seen. Next, even if the probe position is constant, the surrounding tumor is growing and the tumor microenvironment might be changing during tumor development. That is why data interpretation needs to be limited to probe localization, rather than generalized to the whole tumor. To sum up, data interpretation can be challenging, especially for heterogeneous tissue.

## Materials and methods

### Tumor models

Both 4T1 mouse mammary carcinoma cells, and B16F10 melanoma cells line, purchased from the American Type Culture Collection (ATCC), and B16F10 (ATCC) cells were grown at 37°C in a humidified atmosphere of 5% CO2/95% air in RPMI 1640 containing 10% heat-inactivated fetal bovine serum plus 1% penicillin-streptomycin under sterile tissue culture conditions.

4T1 cells were inoculated into mammary fat pad near inguinal glands of BALB/c female mice (N = 39) at the age of 3 months. Mice were obtained from the animal breeding facility at the Medical University of Bialystok (Bialystok, Poland). B16F10 cells were inoculated intradermally in C57BL/6J, male mice (N = 29) at the age of 3 months were obtained from the animal breeding facility at the Faculty of Biochemistry, Biophysics and Biotechnology of the Jagiellonian University (Cracow, Poland). Both tumortypes were located in orthotopic positions. Animal experiments were performed in accordance with EU and national regulations for animal experimentation, Local Committee for Animal Research approval no 76/2017 and 223/2018. Mice were housed in standard laboratory conditions LD:12/12, humidity: 60%, temperature: 23°C. Standard chow diets with free access to drinking water were given in community cages.

For each BALB/c mouse 5x10^5^ 4T1 cells, suspended in 50 μl of PBS, were injected into the mammary fat pad. For each C57BL/6J mouse 1x10^5^ B16F10 cells, suspended in 100 μl of Matrigel mixed with 50 μl PBS, were injected intradermally into the right flank. In one group of the C57BL/6J mice (N = 18), the OxyChip probe was inoculated together with the cells; in the second group (N = 11), the probe was inoculated into the already formed tumor, about 3 mm in size. Only implanting spin probe together with tumor cells allow us to study very small tumors and obtain tissue oxygenation information before ITPP treatment. During our study, for ethical reasons, we decided to start ITPP treatment when the tumors were <100 mm^3^, however, some animals had to be euthanized during the experiment because of the tumor volume (as an outcome, the number of animals in the study is changing). A different number of animals in the groups reflects problems with hypoxia prediction in the tumor tissues–it is impossible to predict if probe localization will be normoxic or hypoxic and studied tumors have extensive intra- and inter-tumoral oxygen heterogeneity. According to radiological definition, the normoxic tumor area was defined as pO_2_ >10 mm Hg, whereas hypoxic areas was defined as pO_2_<10 mm Hg. The impact of oxygen on the response of the tumors can be characterized as a continuous function, however the oxygen enhancement effect is only significant for tissue pO_2_ below 10 mm Hg, and that is why to simplify our analysis we decided to use 10 mm Hg as a threshold [32].

### ITPP treatments protocols

ITPP was a kind gift from Professor J-M Lehn (Institut de Science et d’Ingenierie Supramoleculaires, Universite de Strasbourg, Strasbourg, France). The solution was prepared according to Kieda et al. [17].

Before ITPP treatment, animals were randomized into two groups (ITPP or saline solution). In one set of experiments (4T1 tumors, mice injected with 3 ITPP doses), animals were additionally randomized based on preliminary pO_2_ level in the tumor, to equalize normoxic and hypoxic tumors population.

Four different protocols of ITPP treatments were studied. 4T1 tumors were subjected to either 3 (8, 12 and 16 days after tumor inoculation, 1.5 g/kg, IP) or 4 (8, 9, 15, 16 days after tumor inoculation, 1.5 g/kg, IP) doses of ITPP. B16F10 tumors were treated with 6 ITPP injections on either 5, 6, 11, 12, 17, 18 or 5, 6, 12, 13, 19, 20 days after tumor inoculation, all as an intraperitoneal dose of 2 g/kg. These treatment protocols were designed based on earlier results by Tran et al. [33] and pilot experiments. The treatment schedules were adjusted to the tumor growth rate.

Mice were randomized into two groups based on ITPP or saline (control, vehicle for ITPP) administration for each protocol: 4T1 tumors treated with 3ITPP doses: N = 10 for ITPP and N = 10 for control; 4 ITPP doses protocol N = 10 for ITPP group and N = 9 for saline-treated animals; for B16F10 tumors received six doses at day 5,6,11,12,17,18 there were N = 11 for ITPP and N = 7 for the control group; second 6 dose protocol (injections at day 5,6,12,13,19,20) has N = 6 for ITPP group and N = 5 for saline-treated animals.

### Tumor oxygenation measurements

In vivo EPR measurements were conducted at L-band (Bruker Elexsys-II E540, Germany) CW EPR spectrometer using a surface coil (Bruker, Germany). Anesthesia was induced by 3 vol% isoflurane (Aerrane, Baxter Polska Sp. z o. o., Poland) and then maintained at 1.5–2.0 vol% isoflurane in air, delivered at 1.2 l/min via the nose cone. Mouse breathing rate and temperature were monitored.

In vivo EPR spectroscopy was conducted with the following parameters: microwave power = 10.75 mW; center field = 389 G; modulation frequency = 100 kHz; modulation amplitude = 0.18 G. Single imaging session provides an average EPR signal from et least 5 spectras. OxyChip (a kind gift from Prof. Perianann Kuppusamy, Geisel School of Medicineat Dartmouth College (Hanover, New Hampshire, USA) was used as an oxygen-sensitive spin probe [51,52]. 1 mm piece of Oxychip was implanted in tumor tissue using a 22G needle at least three days before EPR measurements to allow healing. Implantation occurred on the inoculation day or the eighth day of tumor growth for B16F10 tumors and the four days of tumor growth for 4T1 tumors. OxyChip spin probe, coupled with spectroscopic EPR measurements, allows getting information about mean oxygen concentration (calculated from spectrum line width based on calibration curve) in a tumor area around the implantation site. Localization of the crystals in the tumor as confirmed using ultrasound (B-mode, description at 2.4), or during necropsy. EPR measurements were performed before and 24 hours after each ITPP dose. Multiple EPR signals (between 2–6) were collected at selected time points, depending on the signal-to-noise ratio, stability of anesthesia, and signal consistency.

Our previous study did not find any relationship between the depth of solid spin probe implantation (measured from the skin to probe) and measured oxygen partial pressure [34].

### Ultrasound measurements

A high-resolution ultrasound (US) imaging system designed for small experimental animals (Visual Sonics Vevo 2100, Canada) with an MS-550D transducer with a center operating frequency of 22–55 MHz was used for ultrasound measurements of 4T1 tumors. During imaging, body temperature was controlled by the heating pad and maintained at 37°C. Anesthesia was induced by 3 vol% isoflurane (Aerrane, Baxter Polska Sp. z o. o., Poland) and then maintained at 1.5–2.0 vol% isoflurane in air, delivered at 1.2 l/min via a nose cone. Ultrasonographic imaging of tumors was conducted on the same day as EPR oximetry. For the initial confirmation of the tumor, a B-mode ultrasound (greyscale) was performed with a central frequency of 40 MHz. Doppler imaging is used in ultrasound to detect the presence of blood flow and to evaluate the direction and speed of flow in vessels. Power Doppler (PD) measurements were performed with a central frequency of 32 MHz and pulse repetition frequency (PRF) 3–4 kHz. 3D images of tumors, each containing about 40 scans per tumor, were obtained with a 0.2 mm step using a steady-arm-held transducer. The PD measurements were performed only on one of the most promising ITPP protocols (4T1 tumor model with three ITPP/saline doses at day 8,12,16), due to prolong anesthesia during the multi-imaging session.

### EPR and US data analysis

EPR data analysis was performed with the use of custom MATLAB (Mathworks, USA) scripts. Recorded EPR spectra were fitted using EasySpin toolbox, and the spectrum line width was measured. Oxygen concentration was calculated from the spectrum line width based on the calibration curve determined in buffer solution bubbled with a range of oxygen concentrations. Ultrasound data analysis was performed in Vevo LAB 3.2.0 (VisualSonics®, Canada) software. From each image, blood flow voxels within the tumor border were counted. Tumor borders were delineated by hand in all B-mode image slices. The tumor volume and vascularity (percent of functional vessel volume in tumor volume, VP) were calculated.

### Data post-processing and statistical analysis

**Fig 1:**
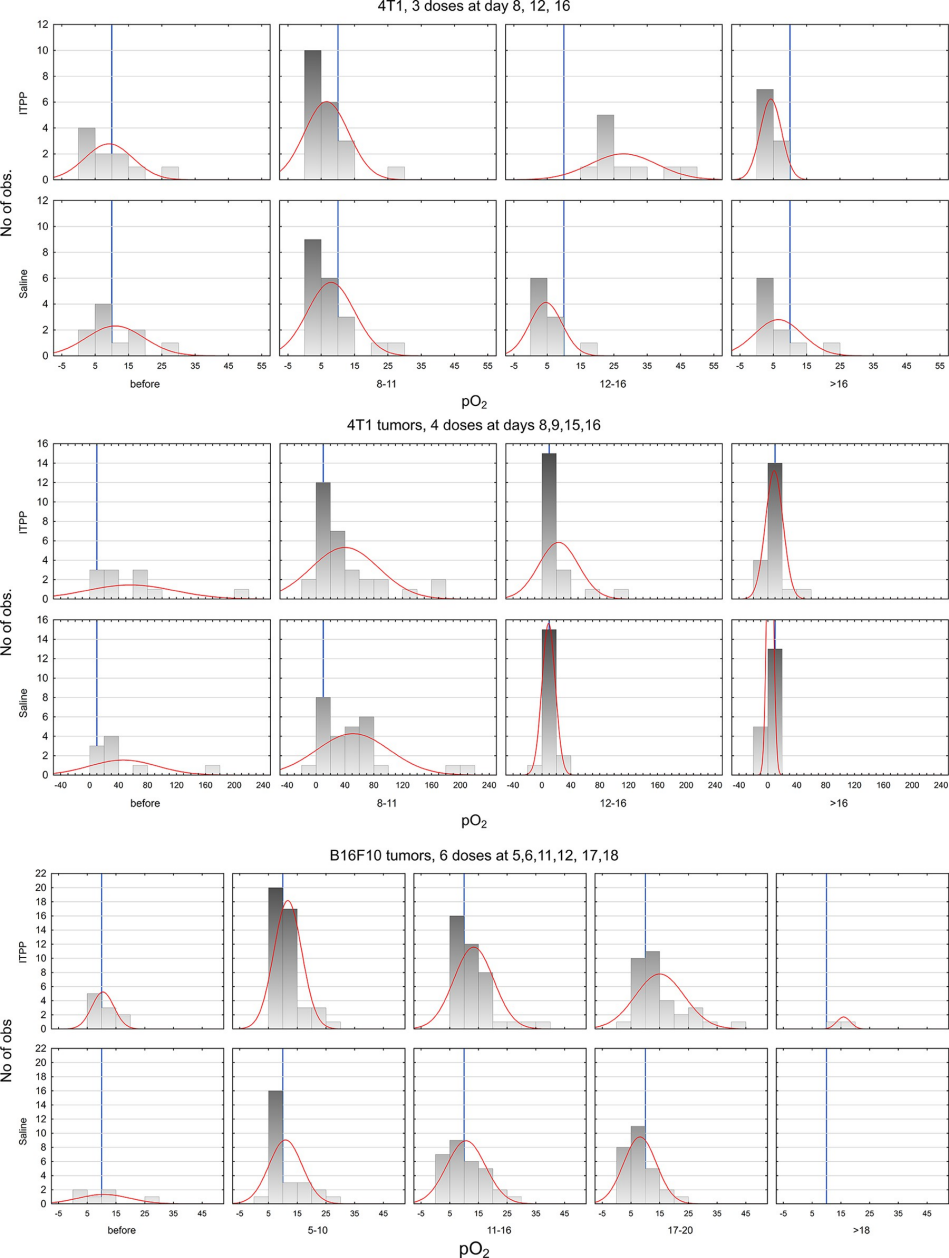
Histograms of tumors pO_2_ at different time points. Histograms of pO_2_ [mm Hg] data with Gaussian fit (red). B16F10 tumors oxygenation data are presented in **A** and **B** both protocols contained 6 ITPP doses but with different time regiment, **A**: 5,6,11,12,17 and 18 days post tumor inoculation, **B**: 5,6,12,13,19,20 days post tumor inoculation. Data from 4T1 tumors are presented in **C**: 3 ITPP doses were administrated at 8,12 and 16 days post tumor inoculation, **D**: 4 ITPP doses administrated at 8,9,15,16 days post tumor inoculation. Time points after ITPP/saline injection were categorized in following periods for 4T1 tumors 8–11, 12–16 and >16 days and for B16F10 tumors 5–10, 11–16, 17–20 and >20 days after tumor inoculation. Due to different setup of experiments only one treatment protocol includes information of B16F10 tumor oxygenation before first ITPP/saline dose (see methods). The blue line marks 10 mm Hg. Data were collected from 39 BALB/c mice and 29 C57BL/6J mice.

To better understand ITPP effects on tissue oxygenation, tumors were classified based on local pO_2_ values before treatment as normoxic (>10 mm Hg) or hypoxic (<10 mm Hg) (Figs 4 and S2). Additionally, to investigate changes in tumor oxygenation during the experimental time, each local pO_2_ value for the selected time point was classified as normoxic and hypoxic (Figs 1 and S1).

**Fig 2:**
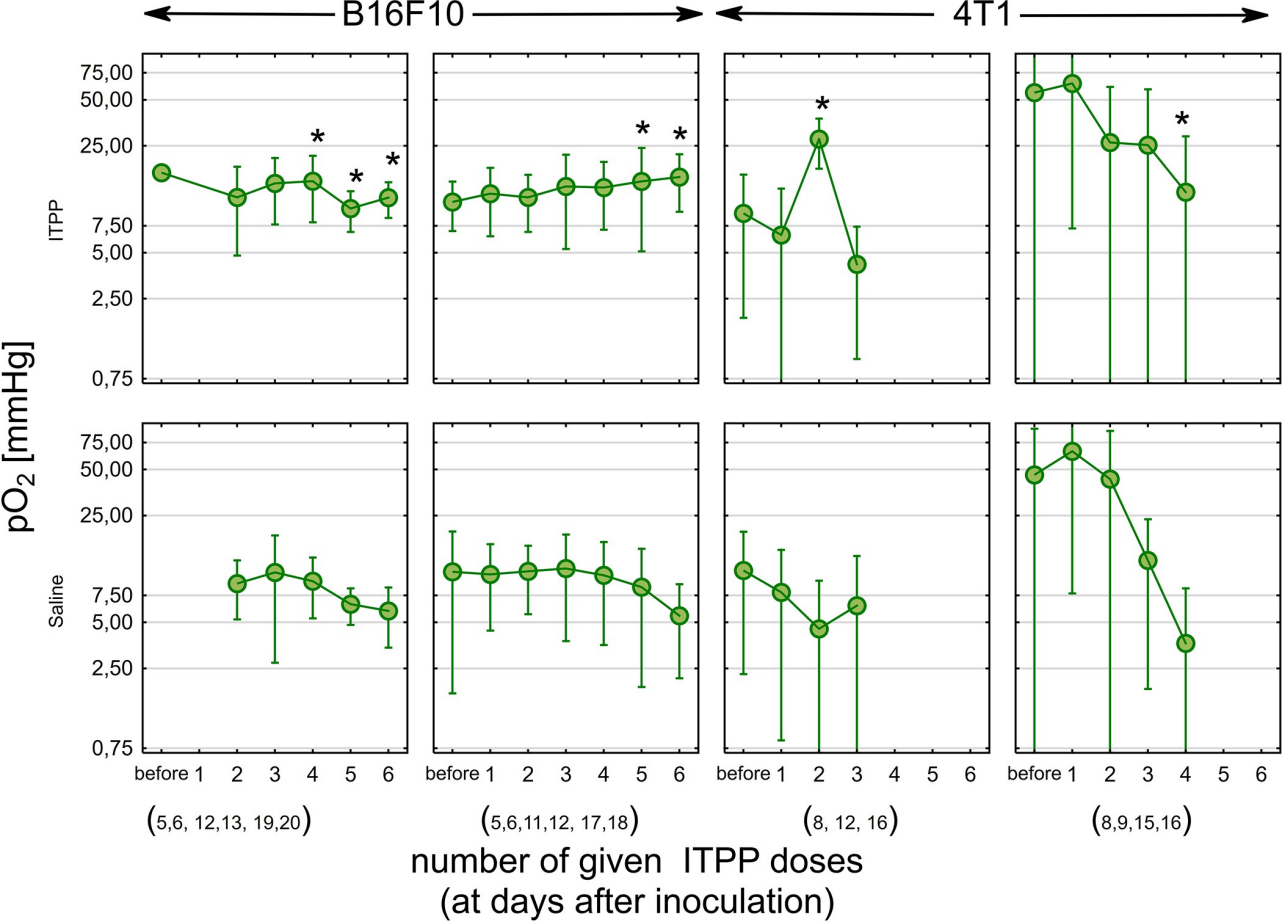
pO_2_ changes during ITPP treatment. Local pO_2_ measurements in B16F10 tumors (6 ITPP doses injected at 5,6,11,12,17,18 or 5,6,12,13,19,20 days after tumor inoculation) and 4T1 (ITPP injected at 8,12,16 or 8,9,15,16 days after tumor inoculation). All points represent the mean pO_2_ level with SD for all EPR measurements performed after 1–6 ITPP doses (depending on the protocol). The time gap between ITPP injections was between 1–6 days. * p<0.05 based on a comparison between saline and ITPP-treated mice after selected conditions (Kruskal-Wallis ANOVA). The same data as presented in Fig 1, collected from 39 BALB/c mice and 29 C57BL/6J mice. Due to the inability to collect data in early time points for one group of B16F10 tumors, two points are missing (before and after 1 dose of ITPP).

**Fig 3:**
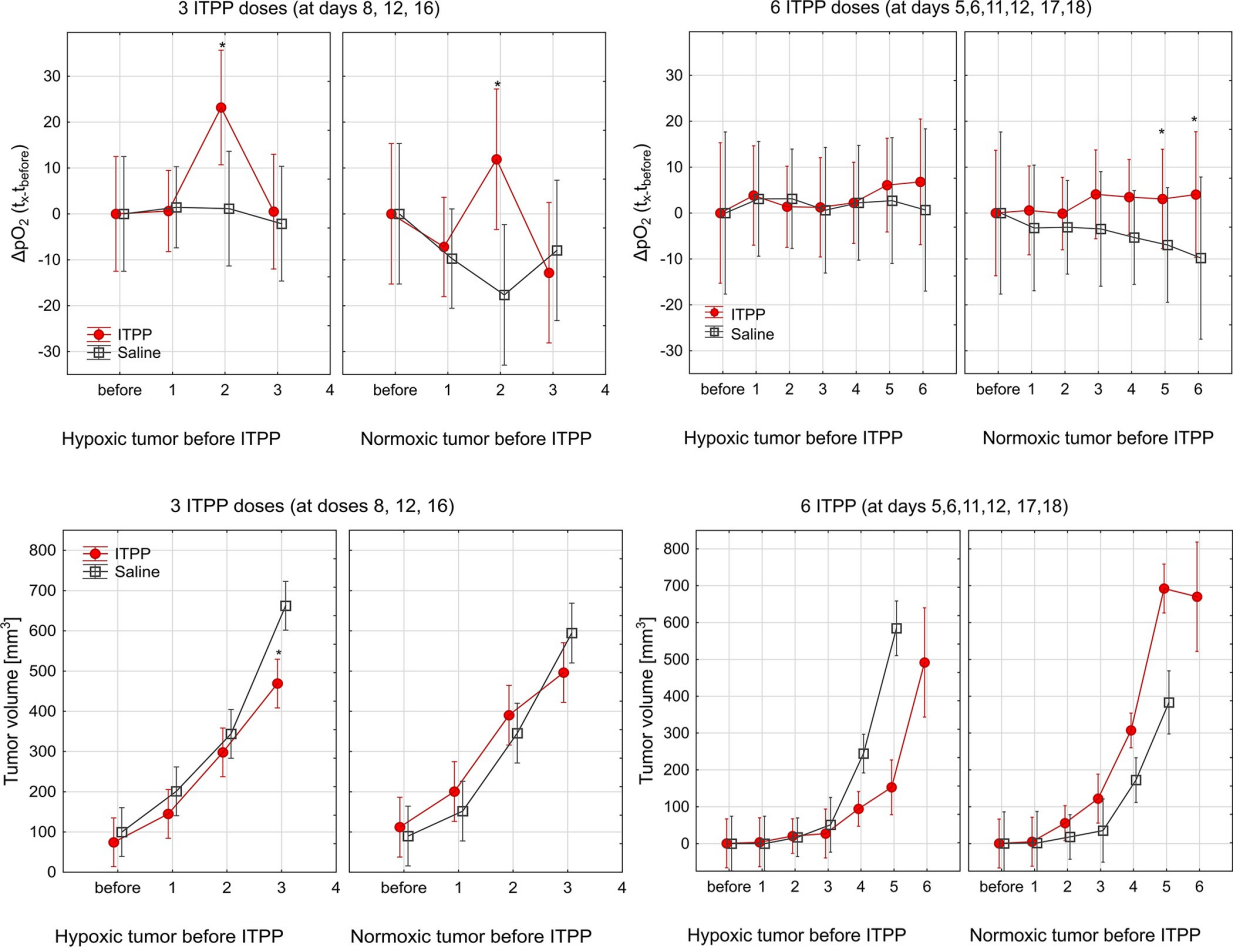
Relative changes of pO_2_ during ITPP treatment. (A, B) Change of pO_2_ level (ΔpO_2 tx-tbefore_, for each tumor) and (C, D) tumor growth kinetic of 4T1 and B16F10 tumors. Breast cancer tumors were treated with 3 ITPP injections at 8,12, and 16 days after inoculation, whereas melanoma-bearing mice received 6 ITPP injections at 5,6,11,12,17, and 18 days after tumor inoculation. All pO_2_ data are represented as means with standard errors as a function of number of the ITPP dose (time gaps between doses were from 1–6 days). All 4T1 (A, C) and B16F10 (B, D) tumors were divided into normoxic and hypoxic before treatments. Marked significance only between ITPP and saline treated animals with hypoxic or normoxic tumors, * p≤0.05 (Kruskal-Wallis ANOVA). Additional statistical significance is described in the text. As presented in Fig 1, the same data were collected from 20 BALB/c mice and 18 C57BL/6J mice.

**Fig 4:**
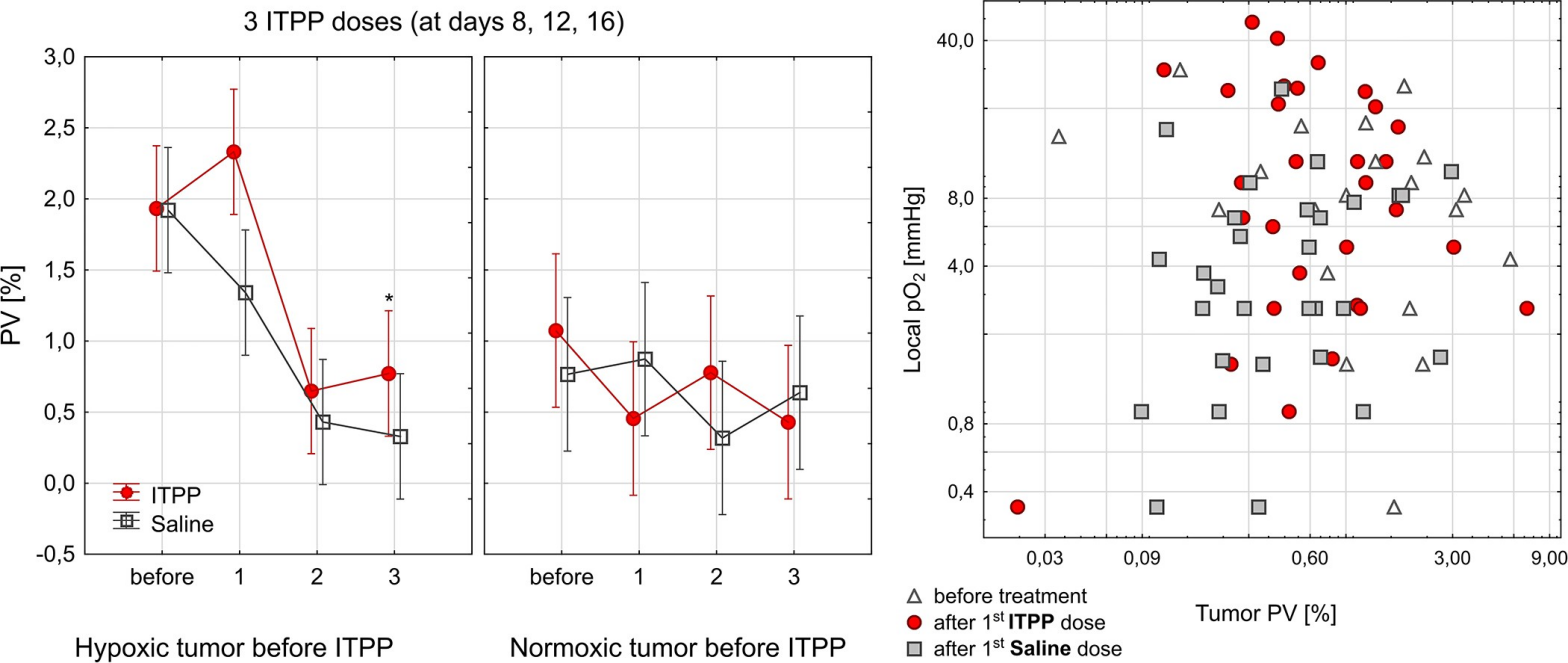
Blood vessels dynamic during ITPP treatment. (A) Doppler detected volume of active blood vessels expressed as a percent of tumor volume (PV) in normoxic and hypoxic 4T1 tumors after treatment with three ITPP/Saline doses (number of doses presented on the x-axis), data points represent means ± standard errors. (B) Local pO_2_ did not correlate with tumor PV, neither for tumors before treatment nor after ITPP/Saline injections. Linear correlation statistics—before treatment: r = -0.09, p = 0.47, r^2^ = 0.01; after 1^st^ ITPP dose: r = -0.23, p = 0.52, r^2^ = 0.05; after 1^st^ saline dose: r = -0.44, p = 0.2, r^2^ = 0.2. Data were collected from 20 BALB/c mice.

Because local pO_2_ data were collected for a prolonged time and at different time points were collected between ITPP treatment protocols, to allow data comparison between groups, pO_2_ values were categorized into the following periods: before injections, 8–11, 12–16, and >16 days after 4T1 tumor inoculation; before injections, 5–10, 11–16, 17–20 and >20 days after B16F10 tumor inoculations (Figs 1 and S1). To highlight the influence of each given dose, some analyses were performed as a function of the number of injections (Figs 2–4, S2 and S3). Data was analyzed in Statistica13® software with multifactorial and repeated measurements ANOVA to determine significant effects of factors like ITPP or tumor oxygenation status. For pair analysis, Kruskal-Wallis ANOVA was used. P<0.05 was considered statistically significant. The same pO_2_ experimental data from tumors is presented differently in Figs 1–3 and S1–S3.

Raw data are accessible via Jagiellonian University repository (RODBUK) with DOI number: doi:10.57903/UJ/K3QDYS.

## Results

### Tumors become more normoxic after ITPP treatment

Intertumoral variability in pO_2_ values was much higher in untreated 4T1 than B16 tumors. The mean and SD values were 27.75±33.23 mm Hg for all the 4T1 tumors pre-treatment and 10.72±6.09 mm Hg for B16F10 tumors. This high heterogeneity is evident in wide histograms in Fig 1. A threshold of 10 mmHg (blue line) was introduced, following the traditional radiobiological definition of tumor hypoxia, useful in the context of therapies [12]. Each local pO_2_ value could qualify this particular tumor area as hypoxic (pO_2_<10 mm Hg) or normoxic (pO_2_>10 mm Hg) at the measurement time. Such thresholding permits an easy following of tumor hypoxia fluctuations over time (Figs 1 and S1). ITPP induced substantial changes in tumor oxygenation (Figs 1 and 2). Hypoxia was alleviated in two different immunocompetent orthotopic tumor models, either transiently or persistently. Tumor oxygenation status changes depending on the tumor type and treatment protocols used. In B16F10 tumors, 50% of the examined areas before the first ITPP injection are below 10 mm Hg. After ITPP treatment, B16F10 tumors are always more normoxic than the control for each time period. Even 100% of normoxic tumor areas were achieved in the last period after 6 ITPP doses (at days 5,6,11,12,17, and 18 post-tumor inoculations) (Fig 1A). A slightly different regime of 6 ITPP injections (ITPP injected at days 5,6,12,13,19 and 20) resulted in a similar pattern (Fig 1B). Generally, for B16F10 tumors, long-term treatment with ITPP led to a more normoxic tumor status. Comparable trends were observed for 4T1 tumors (Fig 1C and 1D). Animals treated with saline developed tumors with more than 75% hypoxic areas in the late period (>16 days post inoculation). A transient pattern of increased oxygenation was observed in BALB/c mice treated with three-dose ITPP protocol (Fig 1C), i.e. 100% of tumor areas became normoxic between days 12 and 16 but later on (past 16 days) 100% areas became hypoxic again (the quantitative data are presented as histograms in S1 Fig). The four-dose ITPP protocol also caused tumor areas to be more normoxic, especially after the third ITPP dose (Fig 1D). It should be pointed out that 4T1 tumors grow very rapidly and that oxygenation of all the 4T1 tumors in Fig 1D were much more heterogeneous, possibly due to their smaller size at the start of the experiment (<100 mm^3^).

### ITPP induces the oxygen therapeutic window depending on tumor model and treatment procedure

The time kinetics of mean pO_2_ levels strongly depended on tumor type and treatment protocol (Fig 2). The highest pO_2_ increase related to ITPP administration was observed for 4T1 tumors after the three-dose ITTP treatment protocol. A lower but more sustained increase in local pO_2_ was seen in C57BL mice treated with 6 ITPP dosses, especially in the second six-dose ITTP protocol (injection days 5,6,11,12,17,18). As expected, oxygen partial pressure decreased in the control 4T1 and B16F10 tumors over time. For all the tested treatment protocols, before the treatment, the mice did not have significantly different local tumor oxygenation (0.55<p≤1). It should be highlighted that oxygenation of 4T1 tumors is more heterogeneous, as demonstrated by the high standard deviation (SD) of local pO_2_ compared to B16F10 tumors. In addition, pO_2_ data were not collected for C57BL/6J mice after the sixth saline dose due to the loss of mice for ethical reasons.

A significant effect was observed in B16F10 tumors treated with 6 ITPP doses at days 5,6,11,12,17 and 18 post-tumor inoculation, where local pO_2_ levels increased after dose five to 14.6±9.55 mm Hg, whereas in control animals, it was 8.5±6.61 (p = 0.024). Similarly, after the sixth ITPP dose, local pO_2_ was 15.6±6.38 mm Hg for ITPP-treated animals and 5.5±3.37 mm Hg for saline-injected mice (p<0.002). A significant increase in local pO_2_ level was also observed in B16F10 tumors treated with the second ITPP protocol (6 injections at days 5,6,12,13,19, and 20 post-tumor inoculations). After the fourth ITPP dose the increase of pO_2_ was from 9.3±3.97 mm Hg for saline-treated animals to 15.2±6.93 mm Hg after ITPP (p = 0.009). Despite the slight decrease in pO_2_ in the ITPP-treated group (9.7±2.9 mm Hg after the 5th ITPP dose and 11.44±2.99 mm Hg after dose six), in the controls, pO_2_ was much lower (5.9±2.5 mm Hg after six saline injections, p = 0,04 after dose five and p = 0,02 after dose six). In B16F10 tumors, in comparison to 4T1, the pO_2_ increase was not as high and was achieved late in the tumor growth, i.e., in old tumors.

In contrast, a considerable increase of local pO_2_ in 4T1 tumors was observed already after the second ITPP dose, for BALB/c mice treated with the three-dose ITPP protocol. The observed maximum reached 27.7±9.94 mm Hg (after the second ITPP dose), whereas the local pO_2_ for control tumors was 4.5±4.83mm Hg (p<0.0002). This increase was transient as the third ITPP dose was not associated with any oxygenation difference (4.2±3.19 mm Hg vs. 6.4±7.12 mm Hg, p = 0.67). The four-dose ITPP protocol significantly increased local tumor pO_2_ to 12.4±16.38 mm Hg, whereas in the control mice, the mean pO_2_ was 3.6±4.69 mm Hg (p<0.005).

### Normoxic and hypoxic tumors react differently to ITPP treatment

To monitor the relevance of tumor oxygenation status before the ITPP treatment, tumors were classified as normoxic (>10 mm Hg) or hypoxic (<10 mm Hg) based on local pO_2_ before injections. For control 4T1 and B16F10 hypoxic tumors, the mean change in the local pO_2_ level (ΔpO_2_ = t_x_-t_before_, calculated for each tumor) was close to zero for the observation period. The ΔpO_2_ was introduced because of the high heterogeneity of the population. This was not surprising, as, in already hypoxic tumors, pO_2_ cannot decrease much further. What is more, normoxic control tumors present on average a 10 mm Hg decrease of local pO_2_ during the experimental period (Fig 3A and 3B), (p<0.025). This is an expected effect because as tumors grow, their pO_2_ decreases. Interestingly, an approximately 40 mm Hg decrease was found for the four-dose protocol (S2A Fig). These higher values of ΔpO_2_ confirmed that normoxic 4T1 and B16F10 tumors become more hypoxic due to tumor growth. In comparison, tumors treated with ITPP demonstrate sustained (e.g., Fig 3B) or transient (e.g., Fig 3A) increases in local pO_2_ level. Statistical analysis of pO_2_ as a function of time (repeated measures ANOVA test) revealed that pO_2_ change in ITPP vs. saline group had a high significance both in mice with 4T1 tumors (p<0.00001) and with B16F10 tumors (p<0.05).

Mice with hypoxic 4T1 tumors treated with a three-dose ITPP protocol responded with an average of 23±9 mm Hg increase in local pO_2_ after the second ITPP dose (Figs 3A and S2A), whereas, in normoxic tumors, pO_2_ only increased to 11±14.2 mm Hg at the same time point (p = 0.2). After the third ITPP dose both tumor areas displayed a decrease of the mean pO_2_: 0.5±4.3 mm Hg for hypoxic and -13±7.5 mm Hg for normoxic tumors (p = 0.01). Thus, in normoxic vs. hypoxic 4T1 tumors after ITPP treatment, local pO_2_ was transiently increased but then decreased rapidly (p = 0.01). In the case of B16F10 tumors, however, no significant difference of ΔpO_2_ was found between hypoxic and normoxic B16F10 tumors treated with ITPP, which was probably due to the low number of mice in this group. In both groups, however, a tendency for a slow increase of pO_2_ was observed: 7±6.6 mm Hg for hypoxic and 4±7 mm Hg for normoxic tumors after the last ITPP dose (Fig 3B). Of note are the high spread of the presented values, which indicate heterogeneity of the effects.

The evolution of tumor volume changes after treatment, with tumor oxygenation status before the first dose of saline or ITPP, revealed that hypoxic tumors treated with ITPP grew slightly slower (Fig 3C and 3D). Hypoxic 4T1 tumors treated with ITPP, after the third ITPP dose, were on average smaller by 193 mm^3^ than hypoxic control 4T1 tumors (p<0.04), (Fig 3C). A similar observation was made for hypoxic B16F10 tumors, where hypoxic tumors treated with the five-dose ITPP protocol were on average smaller by 432 mm^3^ than control (p = 0.05) (Fig3D). What is more, normoxic B16F10 tumors were growing a little faster after ITPP treatment (6th dose at days 5,6,11,12,17,18 post-tumor inoculation) in comparison to mice injected with saline (p = 0.08, repeated measures ANOVA) (Fig 3D).

### Hypoxic but not normoxic tumors demonstrate a transient increase in blood flow

To determine how ITPP treatment can modify the tumor vasculature, Doppler ultrasound imaging was used, as it allows the calculation of the percentage of vasculature with the active flow in tumor volume (PV). A slightly higher percentage of the vasculature (1.93±1.55%) was seen in hypoxic 4T1 tumors even before the treatment, in comparison to normoxic tumors with the mean PV of 0.92±0.78 (Fig 4A). However, this was not significant (p = 0.1), perhaps due to the small number of animals. There was a tendency to increased volume of blood flow in tumors that were hypoxic before the treatment: after the first ITPP dose to 2.33±2.45%, whereas in normoxic tumors, PV was 0.45±0.39% (p<0.04). This increase in PV was observed to precede the maximum increase of pO_2_ after the same treatment protocol. After the third ITPP dose in the hypoxic tumor, the PV in ITPP group was significantly higher (p = 0.025) than in the control.

Local pO_2_ did not correlate with the volume of vessels displaying blood flow (Fig 4B).

## Discussion

The selected orthotopic tumor models (B16F10 and 4T1) have heterogeneous oxygenation pO_2_ distribution on three levels: between the tumors, in the tumor tissue, and over time. This high variability is especially important when comparing different tumor models, as the intertumoral heterogeneity in pO_2_ was almost 5.5-fold higher in 4T1 than B16 (based on the comparison of pO_2_ SD before therapy). Additional biological variability was possibly introduced by the separate inoculations (two separate animal series for each treatment protocol). This suggests high biological heterogeneity of the 4T1 tumors, an important factor to take into account when planning experiments. We would like to emphasize that making the assumption that the collected information represents the average of the entire tumor would be unwarranted, as it pertains only to the specific location of the probe. We posit that this may account for the high heterogeneity in our data, as we have observed that intratumoral heterogeneity can exceed intertumoral heterogeneity in our prior experience with 3D tumor imaging. To be able to draw conclusions in such a heterogenous model before and after the treatment, we decided to use three analytical strategies: (i) direct mean pO_2_ analysis (Fig 2), (ii) relative change of pO_2_ for an individual tumor (Fig 3) and (iii) thresholding at 10 mm Hg (Figs 1 and S1). The question can be raised about choice for the threshold value, but we have followed the radiobiological approach and significant radioresistance of cells and tissues below the threshold of 10 mm Hg, which has clinical implications also for other therapies [35]. We have used the same threshold to define hypoxic and normoxic areas (S1 Fig). On one hand, thresholding simplifies our data, but on the other, it allows to predict changes after treatment, especially in combination with radiotherapy.

We decided to adjust the ITPP protocols and OxyChip inoculation technique according to the rate of tumor growth, i.e. the faster B16 tumors require higher ITPP dosing frequency. We did not observe any significant changes in tumor growth kinetics in general, but our study strongly suggests that knowledge about tumor oxygenation prior to the ITPP treatment can answer the question about the possible treatment benefits. In this context, the data obtained by El Hafny-Rahbi et al. [36] can be better understood.

The systemic treatment with ITPP showed an apparent increase in tumor pO_2_, demonstrating that hypoxia in preclinical tumors may be successfully mitigated. The second six-ITPP-dose protocol in B16F10 generated a prolonged oxygen therapeutic window, as the increase of pO_2_ was stable over time (Fig 2). Even though the pO_2_ increase was no greater than 10 mm Hg on average, this is more than enough from the clinical point of view to make tumors more susceptible for radio- or immunotherapy [37]. In contrast, other protocols generated a transient oxygen therapeutic window, even though pO_2_ values increased by 20 mm Hg. A higher ITPP dosing frequency seems to be more effective, as seen by comparing the two six-dose protocols. Although, on average, an increase in local pO_2_ was seen (Fig 2), some tumor areas were not responsive to ITPP (Fig 1). It should be pointed out that a slow but steady increase in pO_2_ seen in old B16 tumors seems to be more promising in terms of stable vessel normalization.

The lower number of ITPP doses and the rapid growth might be responsible for the transient pattern of pO_2_ increase in 4T1 tumors. There are some known differences between these tumor models. B16F10 murine melanoma is a widely applied preclinical model, fast-growing, and known to be very hypoxic. The pO_2_ was around 2–3 mm Hg in subcutaneous B16 tumors [38]. Correspondingly, as seen in Fig 1A, approximately 50% of the tested areas in tumors before the treatment are hypoxic, despite their small volume. On the other hand, 4T1 mammary carcinoma is a model of a triple-negative breast cancer, known for its spontaneous metastases [39,40]. A relatively rich vascular network is present in young tumors, deteriorating quickly with tumor growth. Even though pO_2_ in young 4T1 tumors may be high (Fig 2), it declines rapidly, rendering the tumors hypoxic [33,41]. The ITPP dosing frequency needs to be adjusted to tumor growth rate, with shorter intervals in fast-growing tumors.

An increase of pO_2_ after ITPP was not only seen in hypoxic but also in normoxic areas of the tumors. In B16F10 tumors, the effect was the same in hypoxic and normoxic areas, whereas in 4T1, the increase in pO_2_ was lower in normoxic areas than in the hypoxic areas (p<0.05 after the 1st and 3rd ITPP doses, hypoxic vs. normoxic areas). Previous data has demonstrated that ITPP elevates pO_2_ in hypoxic tumor sites, but it does not do so in normoxic muscle [17]. To clarify the effect of ITPP on normoxic tissues, further experiments are needed.

The vascular function as measured by blood flow (Fig 4A) remained nearly unchanged after ITPP. It has to be kept in mind that our Power Doppler US does not detect microvessels, but more extensive vasculature, of more than 50 μm (based on transducer frequency). All the tumors that were hypoxic before the treatment had higher PV than normoxic at the early stage, which is representative of an overabundant and inefficient vascular network in hypoxia. The tendency to increased PV was seen after the 1st ITPP dose in these tumors, followed by a pO_2_ increase after the second dose (Fig 4A), which suggests that some vascular remodeling was taking place. In general, local pO_2_ did not correlate with PV (Fig 4B), again, being symptomatic of the overall inefficiency of tumor vasculature.

Our study results are similar to those obtained by Tran et al. [33], who measured pO_2_ in six murine and two rat tumor models, and demonstrated an increase in tumor pO_2_ by 7–10 mm Hg for up 72–96 h with slightly different ITPP protocols. They also demonstrated radiosensitization to single-dose radiation, similar to Grgic et al., who demonstrated increased DNA damage after ITPP and radiation [42]. In earlier research, Kieda et al. showed a stable increase in pO_2_ for up to 72 h after two ITPP injections in B16 and 4T1 tumors [17]. However, our data show the possibility for long-term hypoxia mitigation, which may have implications beyond radiotherapy. The protocols of ITPP delivery, adjusted to tumor models, may generate oxygen therapeutic windows in other preclinical experiments.

Several approaches to improving tumor oxygenation have been tried over the years but with little success [43]. One of the most straightforward ones involves systemic oxygen breathing. It is known, however, to lead to vascular contraction, which makes it ineffective in increasing tumor pO_2_. Nevertheless, periodic oxygen supplementation over several weeks, such as, e.g., 100% O_2_; 60 min/day for 21 days or similar, has led to a moderate inhibition of tumor growth and enhancement of antitumor therapies in preclinical ovarian, breast cancer, and melanoma models [44–47]. No significant long-term change of tumor pO_2_ was observed.

Oxygen delivery, either systemically or directly into the tumor, has been attempted using perfluorocarbons and nanotechnological techniques [48]. Oxygen microbubbles administered intravenously and then sonicated with an ultrasound pulse in the tumor provide another way of delivering molecular oxygen directly to the tumor tissue. Oxygen microbubbles have been shown to increase tissue oxygenation and sensitize tumors to radiation [49–53].

The properties that render ITPP capable of increasing pO_2_ inside tumors and any hypoxic site offer new possibilities to revert hypoxia in pathologic tissues during hypoxia-dependent diseases.

The mechanism by which ITPP helps Hb to release its bound oxygen results in a rapid pO_2_ increase that can be observed in shortly after injection. It was demonstrated that BAND 3 protein in red blood cells, which destabilizes glycolytic enzymes, participates in lowering the pH inside the red blood cells, interacts with ITPP and helps release oxygen [15]. The structure of ITPP and its effects on the molecular modifications of endothelial cells in the vessels upon such treatment prompted the hypothesis of its interaction with phosphatases, which, like PTEN, are the primary regulators of angiogenesis. As PTEN is the tumor suppressor that controls the growth of the endothelial cells both at the level of the tip cells where it regulates the Notch DLL signaling for stalk cells as well as their growth of the latter by regulation of the PIK3/AKT/mTOR pathway, it appears as the target for stable vessels normalization. Both mechanisms cooperatively participate in modifying the angiogenic status since PTEN activity is sensitive to hypoxia/reoxygenation with essential effects on the regulation of the microenvironment status [54–56].

## Conclusions

Observed changes in local tumor oxygenation after ITPP treatment indicate two patterns of pO_2_ increase–transient or sustained. Which pattern will be observed depends on ITPP delivery schedule, primarily on the intervals between ITPP doses and the numbers of ITPP doses in relation to the tumor growth rate. It was shown that ITPP treatment could generate a therapeutic oxygen window by shifting hypoxic tumor areas to become normoxic. This strategy may be successfully used for anti-tumor therapies influenced by hypoxia, such as radio-, photo- or immunotherapy. Tumors hypoxic and normoxic before therapy present differences in responding to ITPP treatment. As expected, saline-treated animals with normoxic tumors exhibit a decrease in local tumor oxygenation, which means tumors become more hypoxic over time. At the same time, hypoxic tumors treated with ITPP grew slower than tumors from the control group treated with saline. Moreover, normoxic tumors from mice treated with ITPP may present accelerated growth.

## Supporting information

S1 Fig Pie chart of hypoxic (H, dark blue) and normoxic (N, light blue) tumor areas before and after ITPP treatment. Data from all the All pO_2_ data collected at a particular time point for each protocol were thresholded as hypoxic (<10 mm Hg) or normoxic (>10 mm Hg). B16F10 tumors oxygenation data are presented in A and B; both protocols contained 6 ITPP doses but with different time regiments, A: 5,6,11,12,17 and 18 days post tumor inoculation, B: 5,6,12,13,19,20 days post tumor inoculation. Data from 4T1 tumors are presented in C: 3 ITPP doses were administrated at 8,12 and 16 days post tumor inoculation, D: 4 ITPP doses administrated at 8,9,15,16 days post tumor inoculation. Time points after ITPP/Saline injection were categorized in the following periods for 4T1 tumors 8–11, 12–16 and >16 days and for B16F10 tumors 5–10, 11–16, 17–20 and >20 days. 1B - Due to the different setup of experiments, only one treatment protocol has information on B16F10 tumor oxygenation before the first ITPP/Saline dose (see methods 2.1 and 2.3). 1A –no pie chart for saline >20 due to animals sacrificed related to tumor size. Numbers of animals in the study—A: ITPP N = 11, saline N = 7; B: ITPP N = 6, saline N = 5; C: ITPP N = 10, saline N = 10; D: ITPP N = 10, saline N = 9. The numbers of pO_2_ measurement session used in the study (n) is presented closed to the pie charts symbols H or N. Data collected from 39 Balb/c mice and 29 C57BL/6J mice.
(TIF)

S2 Fig Change of pO_2_ and 4T1 tumor volume after ITPP protocol with 4 doses at days 8, 9, 15 and 16. (A) Change of pO_2_ level (ΔpO_2_ t_x-_t_before_, for each tumor) and (B) tumor kinetic of 4T1 tumors. All pO2 data are represented as means with standard errors as a function of ITPP doses (time gaps between doses were from 1–6 days). Tumors were divided into normoxic and hypoxic before treatments. Marked significance only between ITPP and Saline treated animals with hypoxic or normoxic tumor, * p≤0.05 (ANOVA Kruskala-Wallisa). Additional statistical significance described in the text. Data collected from 19 Balb/c mice.
(JPG)

S3 Fig Average tumor volume kinetics for ITPP (first row) and saline (second row) groups. Measurements in B16F10 tumors (6 ITPP doses injected at 5,6,11,12,17,18 or 5,6,12,13,19,20 days after tumor inoculation) and 4T1 (ITPP injected at 8,12,16 or 8,9,15,16 days after tumor inoculation). All points represent mean tumor volume with SD for all measurements performed after 1–6 ITPP doses (depends on protocol). Time gap between ITPP injections was between 1–6 days. * p<0.05 based on comparison between saline and ITPP treated mice after selected conditions (Kruskal-Wallis ANOVA). The same data as presented in Fig 1, collected from 39 Balb/c mice and 29 C57BL/6J mice.
(JPG)

## References

[1] Vaupel P., Mayer A., Hypoxia in cancer: significance and impact on clinical outcome, Cancer Metastasis Rev. 26 (2007) 225–239. doi: 10.1007/s10555-007-9055-1 17440684

[2] Semenza G.L., Hypoxia and cancer, Cancer Metastasis Rev. 26 (2007) 223–224. doi: 10.1007/s10555-007-9058-y 17404692

[3] Dewhirst M.W., Relationships between Cycling Hypoxia, HIF-1, Angiogenesis and Oxidative Stress Mark, Radiat. Oncol. 172 (2009) 653–665. 10.1667/RR1926.1.Relationships. PMC2790140 19929412

[4] Overgaard J., Horsman M.R., Modification of hypoxia-induced radioresistance in tumors by the use of oxygen and sensitizers, Semin. Radiat. Oncol. 6 (1996) 10–21. doi: 10.1053/SRAO0060010 10717158

[5] Evans N.T.S., Naylor P.F.D., The Effect of Oxygen Breathing and Radiotherapy upon the Tissue Oxygen Tension of some Human Tumours, Br. J. Radiol. 36 (1963) 418–425. 10.1259/0007-1285-36-426-418.

[6] Cárdenas-Navia L.I., Yu D., Braun R.D., Brizel D.M., Secomb T.W., Dewhirst M.W., Tumor-dependent kinetics of partial pressure of oxygen fluctuations during air and oxygen breathing, Cancer Res. 64 (2004) 6010–6017. doi: 10.1158/0008-5472.CAN-03-0947 15342381

[7] Robinson M.F., Dupuis N.P., Kusumoto T., Liu F., Menon K., Teicher B.A., Increased tumor oxygenation and radiation sensitivity in two rat tumors by a hemoglobin-based, oxygen-carrying preparation, Artif. Cells, Blood Substitutes, Biotechnol. 23 (1995) 431–438. doi: 10.3109/10731199509117959 7493064

[8] Riess J.G., Krafft M.P., Fluorinated materials for in vivo oxygen transport (blood substitutes), diagnosis and drug delivery, Biomaterials. 19 (1998) 1529–1539. doi: 10.1016/s0142-9612(98)00071-4 9794531

[9] Jászai J., Schmidt M.H.H., Trends and Challenges in Tumor Anti-Angiogenic Therapies., Cells. 8 (2019) 7–11. doi: 10.3390/cells8091102 31540455 PMC6770676

[10] Izumi Y., Xu L., di Tomaso E., Fukumura D., Jain R.K., Herceptin acts as an anti-angiogenic cocktail, Nature. 416 (2002) 279–280. 10.1038/416279b. 11907566

[11] Jain R.K., Normalizing tumor vasculature with anti-angiogenic therapy: a new paradigm for combination therapy., Nat. Med. 7 (2001) 987–9. doi: 10.1038/nm0901-987 11533692

[12] Moeller B.J., Richardson R.A., Dewhirst M.W., Hypoxia and radiotherapy: Opportunities for improved outcomes in cancer treatment, Cancer Metastasis Rev. 26 (2007) 241–248. doi: 10.1007/s10555-007-9056-0 17440683

[13] O’Hara J.A., Blumenthal R.D., Grinberg O.Y., Demidenko E., Grinberg S., Wilmot C.M., et al, Response to radioimmunotherapy correlates with tumor po2 measured by epr oximetry in human tumor xenografts, Radiat. Res. 155 (2001) 466–473. 10.1667/0033-7587(2001)155[0466:RTRCWT]2.0.CO;2. 11182798

[14] Tian H., Luo Z., Liu L., Zheng M., Chen Z., Ma A., et al, Cancer Cell Membrane-Biomimetic Oxygen Nanocarrier for Breaking Hypoxia-Induced Chemoresistance, Adv. Funct. Mater. 27 (2017) 1–7. 10.1002/adfm.201703197.

[15] Duarte C.D., Greferath R., Nicolau C., Lehn J.M., myo-Inositol trispyrophosphate: A novel allosteric effector of hemoglobin with high permeation selectivity across the red blood cell plasma membrane, ChemBioChem. 11 (2010) 2543–2548. doi: 10.1002/cbic.201000499 21086482

[16] Limani P., Linecker M., Schneider M.A., Kron P., Tschuor C., Kachaylo E., et al, The Allosteric Hemoglobin Effector ITPP Inhibits Metastatic Colon Cancer in Mice, Ann. Surg. 266 (2017) 746–753. doi: 10.1097/SLA.0000000000002431 28742687

[17] Kieda C., El Hafny-Rahbi B., Collet G., Lamerant-Fayel N., Grillon C., Guichard A., et al, Stable tumor vessel normalization with pO2 increase and endothelial PTEN activation by inositol trispyrophosphate brings novel tumor treatment, J. Mol. Med. 91 (2013) 883–899. 10.1007/s00109-013-0992-6. 23471434 PMC3695680

[18] Sato Y., Persistent vascular normalization as an alternative goal of anti-angiogenic cancer therapy, Cancer Sci. 102 (2011) 1253–1256. doi: 10.1111/j.1349-7006.2011.01929.x 21401807

[19] Schneider M.A., Linecker M., Fritsch R., Muehlematter U.J., Stocker D., Pestalozzi B., et al, Phase Ib dose-escalation study of the hypoxia-modifier Myo-inositol trispyrophosphate in patients with hepatopancreatobiliary tumors, Nat. Commun. 12 (2021). doi: 10.1038/s41467-021-24069-w 34155211 PMC8217170

[20] Kuppusamy P., Sense and Sensibility of Oxygen in Pathophysiology Using EPR Oximetry, in: Berliner L.J., Parinandi N.L. (Eds.), Meas. Oxid. Oxidative Stress Biol. Syst., Springer, 2020: pp. 135–187. 10.1007/978-3-030-47318-1_9. 33411441

[21] Swartz H.M., Williams B.B., Zaki B.I., Hartford A.C., Jarvis L.A., Chen E.Y., et al. Unique Opportunities and Some Challenges., Acad. Radiol. 21 (2014) 197–206. 10.1016/j.acra.2013.10.011. PMC3921887 24439333

[22] Halpern H.J., Epel B.M., Going Low in a World Going High: The Physiologic Use of Lower Frequency Electron Paramagnetic Resonance, Appl. Magn. Reson. 51 (2020) 887–907. doi: 10.1007/s00723-020-01261-7 33776216 PMC7992374

[23] Murugesan R., Cook J.A., Devasahayam N., Afeworki M., Subramanian S., Tschudin R., et al, In vivo imaging of a stable paramagnetic probe by pulsed-radiofrequency electron paramagnetic resonance spectroscopy, Magn. Reson. Med. 38 (1997) 409–414. doi: 10.1002/mrm.1910380309 9339442

[24] Ardenkjaer-Larsen J.H., Laursen I., Leunbach I., Ehnholm G., Wistrand L.-G., Petersson J.S., et al, Agents Intended for Oximetric Imaging, J. Magn. Reson. 133 (1998) 1–12. 9654463 10.1006/jmre.1998.1438

[25] Elas M., Krzykawska-Serda M., Gonet M., Kozińska A., Płonka P.M., Electron paramagnetic resonance imaging-solo and orchestra, in: Med. Imaging Methods Recent Trends, Springer Singapore, Singapore, 2019: pp. 1–42. 10.1007/978-981-13-9121-7_1.

[26] Viswakarma N., Siddiqui E., Patel S., Hameed S., Schreiber W., Swartz H.M., et al, In Vivo Partial Oxygen Pressure Assessment in Subcutaneous and Intraperitoneal Sites Using Imaging of Solid Oxygen Probe, Tissue Eng.—Part C Methods. 28 (2022) 264–271. doi: 10.1089/ten.TEC.2022.0061 35509263

[27] Swartz H.M., Vaupel P., Williams B.B., Schaner P.E., Gallez B., Schreiber W., et al, ‘Oxygen level in a tissue’–What do available measurements really report?, Adv. Exp. Med. Biol. 1232 (2020) 145–153. doi: 10.1007/978-3-030-34461-0_19 31893405

[28] Khan N., Williams B.B., Hou H., Li H., Swartz H.M., Repetitive tissue pO2 measurements by electron paramagnetic resonance oximetry: current status and future potential for experimental and clinical studies., Antioxid. Redox Signal. 9 (2007) 1169–82. doi: 10.1089/ars.2007.1635 17536960 PMC2921178

[29] Schaner P.E., Pettus J.R., Flood A.B., Williams B.B., Jarvis L.A., Chen E.Y., et al, OxyChip Implantation and Subsequent Electron Paramagnetic Resonance Oximetry in Human Tumors Is Safe and Feasible: First Experience in 24 Patients, Front. Oncol. 10 (2020). doi: 10.3389/fonc.2020.572060 33194670 PMC7653093

[30] Hou H., Khan N., Gohain S., Kuppusamy M.L., Kuppusamy P., Pre-clinical evaluation of OxyChip for long-term EPR oximetry, Biomed. Microdevices. 20 (2018) 1–10. doi: 10.1007/s10544-018-0272-x 29549438

[31] Kmiec M.M., Tse D., Mast J.M., Ahmad R., Kuppusamy P., Implantable microchip containing oxygen-sensing paramagnetic crystals for long-term, repeated, and multisite in vivo oximetry, Biomed. Microdevices. 21 (2019). doi: 10.1007/s10544-019-0421-x 31286244

[32] Quintiliani M., The oxygen effect in radiation inactivation of DNA and enzymes, Int. J. Radiat. Biol. 50 (1986) 573–594. doi: 10.1080/09553008614550981 3531055

[33] Tran L.-B.-A., Cao-Pham T.-T., Jordan B.F., Deschoemaeker S., Heyerick A., Gallez B., Impact of myo-inositol trispyrophosphate (ITPP) on tumour oxygenation and response to irradiation in rodent tumour models., J. Cell. Mol. Med. 23 (2019) 1908–1916. doi: 10.1111/jcmm.14092 30575283 PMC6378184

[34] Krzykawska-Serda M., Dąbrowski J.M., Arnaut L.G., Szczygieł M., Urbańska K., Stochel G., et al, The role of strong hypoxia in tumors after treatment in the outcome of bacteriochlorin-based photodynamic therapy, Free Radic. Biol. Med. 73 (2014) 239–251. doi: 10.1016/j.freeradbiomed.2014.05.003 24835769

[35] Vaupel P., Mayer A., Hypoxia in cancer: significance and impact on clinical outcome, Cancer Metastasis Rev. 26 (2007) 225–239. doi: 10.1007/s10555-007-9055-1 17440684

[36] El Hafny-Rahbi B., Brodaczewska K., Collet G., Majewska A., Klimkiewicz K., Delalande A., et al, Tumour angiogenesis normalized by myo-inositol trispyrophosphate alleviates hypoxia in the microenvironment and promotes antitumor immune response, J. Cell. Mol. Med. 25 (2021) 3284–3299. doi: 10.1111/jcmm.16399 33624446 PMC8034441

[37] Dewhirst M.W., Mowery Y.M., Mitchell J.B., Cherukuri M.K., Secomb T.W., Rationale for hypoxia assessment and amelioration for precision therapy and immunotherapy studies, J. Clin. Invest. 129 (2019) 489–491. doi: 10.1172/JCI126044 30614815 PMC6355230

[38] Dings R.P.M., Loren M.L., Zhang Y., Mikkelson S., Mayo K.H., Corry P., et al, Tumour thermotolerance, a physiological phenomenon involving vessel normalisation, Int. J. Hyperth. 27 (2011) 42–52. doi: 10.3109/02656736.2010.510495 21204622 PMC3086848

[39] Yang L., Yong L., Zhu X., Feng Y., Fu Y., Kong D., et al, Disease progression model of 4T1 metastatic breast cancer, J. Pharmacokinet. Pharmacodyn. 47 (2020) 105–116. doi: 10.1007/s10928-020-09673-5 31970615

[40] Pulaski B.A., Ostrand-Rosenberg S., Mouse 4T1 Breast Tumor Model, in: Curr. Protoc. Immunol., John Wiley & Sons, Inc., Hoboken, NJ, USA, 2001: pp. 1–16. 10.1002/0471142735.im2002s39. 18432775

[41] Zhang H., Zhang X., Ren Y., Cao F., Hou L., Zhang Z., An in situ microenvironmental nano-regulator to inhibit the proliferation and metastasis of 4T1 tumor, Theranostics. 9 (2019) 3580–3594. doi: 10.7150/thno.33141 31281499 PMC6587164

[42] Grgic I., Tschanz F., Borgeaud N., Gupta A., Clavien P.A., Guckenberger M., et al, Tumor Oxygenation by Myo-Inositol Trispyrophosphate Enhances Radiation Response, Int. J. Radiat. Oncol. Biol. Phys. 110 (2021) 1222–1233. doi: 10.1016/j.ijrobp.2021.02.012 33587991

[43] Vaupel P., Mayer A., Hypoxia in cancer: significance and impact on clinical outcome, Cancer Metastasis Rev. 26 (2007) 225–239. doi: 10.1007/s10555-007-9055-1 17440684

[44] Hatfield S.M., Kjaergaard J., Lukashev D., Schreiber T.H., Belikoff B., Abbott R., et al, Immunological mechanisms of the antitumor effects of supplemental oxygenation., Sci. Transl. Med. 7 (2015) 277ra30. doi: 10.1126/scitranslmed.aaa1260 25739764 PMC4641038

[45] Gogna R., Madan E., Kuppusamy P., Pati U., Re-oxygenation causes hypoxic tumor regression through restoration of p53 wild-type conformation and post-translational modifications, Cell Death Dis. 3 (2012) e286–7. doi: 10.1038/cddis.2012.15 22419115 PMC3317354

[46] Selvendiran K., Kuppusamy M.L., Ahmed S., Bratasz A., Meenakshisundaram G., Rivera B.K., et al, Oxygenation inhibits ovarian tumor growth by downregulating STAT3 and cyclin-D1 expressions, Cancer Biol. Ther. 10 (2010) 386–390. doi: 10.4161/cbt.10.4.12448 20562529

[47] Mast J.M., Kuppusamy P., Hyperoxygenation as a therapeutic supplement for treatment of triple negative breast cancer, Front. Oncol. 8 (2018) 1–9. 10.3389/fonc.2018.00527. 30524959 PMC6256245

[48] Phung C.D., Tran T.H., Pham L.M., Nguyen H.T., Jeong J.H., Yong C.S., et al, Current developments in nanotechnology for improved cancer treatment, focusing on tumor hypoxia, J. Control. Release. 324 (2020) 413–429. doi: 10.1016/j.jconrel.2020.05.029 32461115

[49] Delaney L.J., Ciraku L., Oeffinger B.E., Wessner C.E., Bin Liu J., Li J., et al, Breast Cancer Brain Metastasis Response to Radiation After Microbubble Oxygen Delivery in a Murine Model, J. Ultrasound Med. 38 (2019) 3221–3228. doi: 10.1002/jum.15031 31124171 PMC7064157

[50] Fix S.M., Papadopoulou V., Velds H., Kasoji S.K., Rivera J.N., Borden M.A., et al, Oxygen microbubbles improve radiotherapy tumor control in a rat fibrosarcoma model–A preliminary study, PLoS One. 13 (2018) 1–18. 10.1371/journal.pone.0195667. PMC5891067 29630640

[51] Eisenbrey J.R., Shraim R., Liu J., Li J., Stanczak M., Oeffinger B., et al, Sensitization of Hypoxic Tumors to Radiation Therapy Using Ultrasound-Sensitive Oxygen Microbubbles, Int. J. Radiat. Oncol. 101 (2018) 88–96. doi: 10.1016/j.ijrobp.2018.01.042 29477294 PMC5886808

[52] Ho Y.J., Chu S.W., Liao E.C., Fan C.H., Chan H.L., Wei K.C., et al, Normalization of tumor vasculature by oxygen microbubbles with ultrasound, Theranostics. 9 (2019) 7370–7383. doi: 10.7150/thno.37750 31695774 PMC6831304

[53] Hysi E., Fadhel M.N., Wang Y., Sebastian J.A., Giles A., Czarnota G.J., et al, Photoacoustic imaging biomarkers for monitoring biophysical changes during nanobubble-mediated radiation treatment, Photoacoustics. 20 (2020) 100201. doi: 10.1016/j.pacs.2020.100201 32775198 PMC7393572

[54] Abou Khouzam R., Brodaczewska K., Filipiak A., Zeinelabdin N.A., Buart S., Szczylik C., et al, Tumor Hypoxia Regulates Immune Escape/Invasion: Influence on Angiogenesis and Potential Impact of Hypoxic Biomarkers on Cancer Therapies, Front. Immunol. 11 (2021) 1–16. doi: 10.3389/fimmu.2020.613114 33552076 PMC7854546

[55] Oknińska M., Zambrowska Z., Zajda K., Paterek A., Brodaczewska K., Mackiewicz U., et al, Right ventricular myocardial oxygen tension is reduced in monocrotaline-induced pulmonary hypertension in the rat and restored by myo-inositol trispyrophosphate, Sci. Rep. 11 (2021) 1–9. 10.1038/s41598-021-97470-6. 34504231 PMC8429755

[56] Majewska A., Wilkus K., Brodaczewska K., Kieda C., Endothelial cells as tools to model tissue microenvironment in hypoxia-dependent pathologies, Int. J. Mol. Sci. 22 (2021) 1–25. doi: 10.3390/ijms22020520 33430201 PMC7825710

