# Supplementary figures and images for: Oxygen therapeutic window induced by myo-inositol trispyrophosphate (ITPP)–Local pO2 study in murine tumors
Source: PLoS One. 2023 May 11;18(5):e0285318. doi: 10.1371/journal.pone.0285318 (PMC10174508; doi:10.1371/journal.pone.0285318)

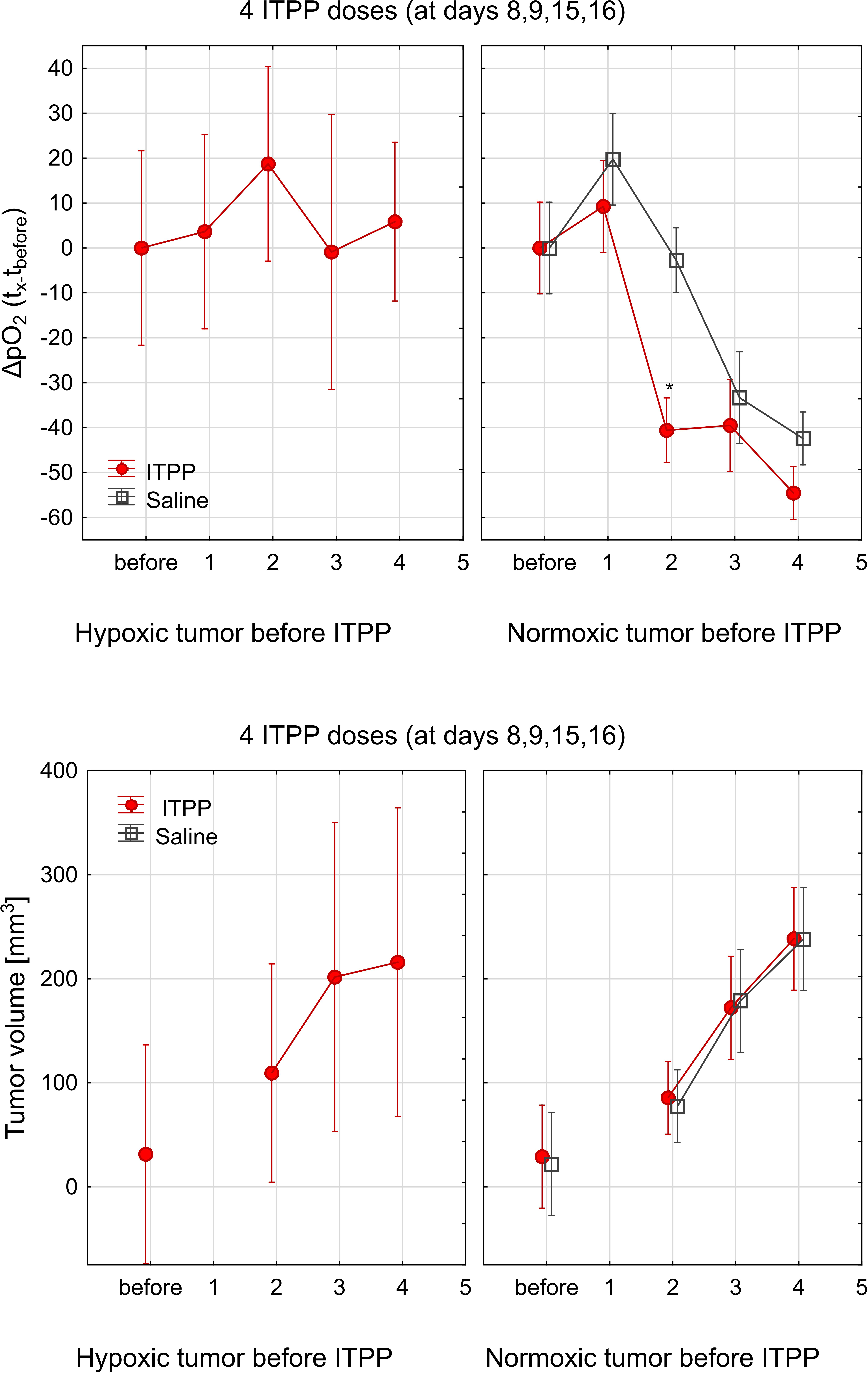

Supplement: S2 Fig — (A) Change of pO2 level (ΔpO2 tx-tbefore, for each tumor) and (B) tumor kinetic of 4T1 tumors. All pO2 data are represented as means with standard errors as a function of ITPP doses (time gaps between doses were from 1–6 days). Tumors were divided into normoxic and hypoxic before treatments. Marked significance only between ITPP and Saline treated animals with hypoxic or normoxic tumor, * p≤0.05 (ANOVA Kruskala-Wallisa). Additional statistical significance described in the text. Data collected from 19 Balb/c mice. (JPG) [file pone.0285318.s002.jpg]

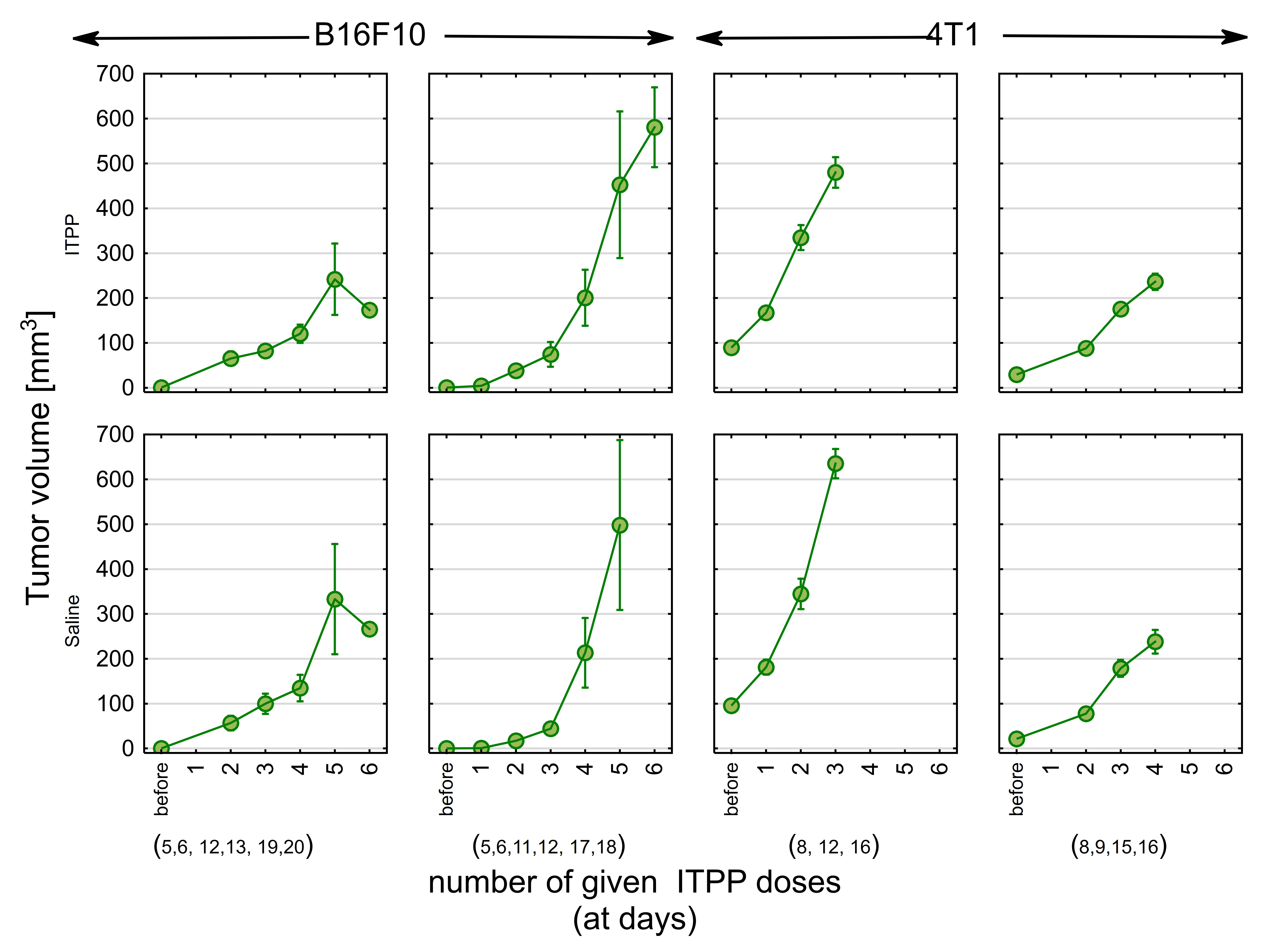

Supplement: S3 Fig — Measurements in B16F10 tumors (6 ITPP doses injected at 5,6,11,12,17,18 or 5,6,12,13,19,20 days after tumor inoculation) and 4T1 (ITPP injected at 8,12,16 or 8,9,15,16 days after tumor inoculation). All points represent mean tumor volume with SD for all measurements performed after 1–6 ITPP doses (depends on protocol). Time gap between ITPP injections was between 1–6 days. * p<0.05 based on comparison between saline and ITPP treated mice after selected conditions (Kruskal-Wallis ANOVA). The same data as presented in Fig 1, collected from 39 Balb/c mice and 29 C57BL/6J mice. (JPG) [file pone.0285318.s003.jpg]
